# Impact of Adherence to the Mediterranean Diet on Antioxidant Status and Metabolic Parameters in NAFLD Patients: A 24-Month Lifestyle Intervention Study

**DOI:** 10.3390/antiox13040480

**Published:** 2024-04-17

**Authors:** Maria Magdalena Quetglas-Llabrés, Margalida Monserrat-Mesquida, Cristina Bouzas, Silvia García, Emma Argelich, Miguel Casares, Lucía Ugarriza, Isabel Llompart, Josep A. Tur, Antoni Sureda

**Affiliations:** 1 Research Group on Community Nutrition & Oxidative Stress, University of the Balearic Islands-IUNICS, 07122 Palma de Mallorca, Spainmargalida.monserrat@uib.es (M.M.-M.); isabel.llompart@ssib.es (I.L.); antoni.sureda@uib.es (A.S.); 2 CIBEROBN (Physiopathology of Obesity and Nutrition), Instituto de Salud Carlos III, 28029 Madrid, Spain; 3 Health Research Institute of Balearic Islands (IdISBa), 07120 Palma, Spain; 4 Radiodiagnosis Service, Red Asistencial Juaneda, 07011 Palma de Mallorca, Spain; 5 C.S. Camp Redó, IBSalut, 07010 Palma de Mallorca, Spain; 6 Clinical Analysis Service, University Hospital Son Espases, 07198 Palma de Mallorca, Spain

**Keywords:** non-alcoholic fatty liver disease, intrahepatic fat contents, lifestyle, erythrocytes, antioxidant score

## Abstract

Background: The Mediterranean Diet (MedDiet) is recognized as a healthy dietary pattern. Non-alcoholic fatty liver disease (NAFLD) is characterized by the excessive accumulation of fat in the liver. Objectives: To assess the antioxidant status in erythrocytes, plasma, and peripheral blood mononuclear cells (PBMCs) of NAFLD patients following a 24-month lifestyle intervention based on the MedDiet. Adult patients (*n* = 40; aged 40–60 years) diagnosed with NAFLD by magnetic resonance imaging were divided into two groups based on their adherence to the MedDiet. Consumption was assessed using a validated 143-item semiquantitative Food Frequency Questionnaire. Anthropometrics, biochemistry parameters, intrahepatic fat contents (IFC), antioxidants, and inflammatory biomarkers were measured in plasma and erythrocytes before and after the intervention. Results: After the intervention, body mass index (BMI) and plasma levels of total cholesterol, low-density lipoprotein cholesterol (LDL-chol), triglycerides, malondialdehyde (MDA), and cytokeratin-18 (CK18) decreased, and high-density lipoprotein cholesterol (HDL-chol) increased. Participants with high adherence to MedDiet showed lower IFC, hepatic enzyme (AST, ALT, and GGT), glycemia, oxidase LDL (oxLDL) plasma levels, and erythrocyte MDA levels. Higher antioxidant activity (erythrocyte catalase-CAT, superoxide dismutase-SOD, glutathione peroxidase-GPx, glutathione reductase-GRd, and total glutathione-GSH as well as PBMCs-CAT gene expression) was observed in these patients, along with a reduction of PBMCs reactive oxygen species production and Toll-like receptor 4 (TLR4) expression. Inverse associations were observed between adherence to the MedDiet and BMI, glycemia, AST, IFC, and CK18 plasma levels and oxLDL, CAT, SOD, and GRd activities in erythrocytes. A significant linear regression was observed between adherence to the MedDiet and antioxidant score. Conclusions: Adherence to the MedDiet is associated with improved plasma and PBMC antioxidant and inflammatory biomarker profiles and high antioxidant defences in erythrocytes.

## 1. Introduction

The Mediterranean diet (MedDiet) is defined as a dietary pattern with a high intake of whole grains, fruits, legumes, vegetables, and nuts. Conversely, it advocates for minimal consumption of meat and poultry, moderate dairy intake, and moderate alcohol consumption, especially when red wine is consumed with meals. A hallmark of the MedDiet is the predominant use of olive oil, renowned for its health-promoting properties, while saturated fatty acids are kept to a minimum [1]. This dietary pattern emphasizes a higher intake of monounsaturated and omega-3 fatty acids and a decrease in carbohydrates, especially those found in sweets and refined products [2]. The MedDiet can be regarded as the benchmark in preventive medicine, addressing a wide range of disorders associated with oxidative metabolism imbalance. Its efficacy stems from the synergistic combination of several foods possessing antioxidant and anti-inflammatory properties, surpassing the impact of any individual nutrient or dietary component [3].

Non-alcoholic fatty liver disease (NAFLD), recently rebranded as metabolic-associated fatty liver disease (MAFLD), has emerged as one of the most prevalent liver diseases globally, escalating alongside the obesity epidemic [4]. An NAFLD diagnosis traditionally requires a liver biopsy, considered the gold standard. However, the chronic nature of this disease presents challenges for conducting periodic biopsies on a large scale [5]. This leads to the use of other techniques, such as magnetic resonance imaging (MRI) or ultrasound [6]. NAFLD is closely linked to metabolic syndrome (MetS) and is typified by high intracellular lipid droplets that surpass 5% when there is no discernible alcohol intake [7]. Excessive fat accumulation in the liver is proposed to increase reactive oxygen species (ROS) production, leading to lipid peroxidation and oxidative stress. The primary consequence of oxidative stress is the oxidation of cellular components, thereby accelerating cell death through necrosis and apoptosis [8]. Notably, oxidative stress plays a crucial role in the progression of liver disease from simple steatosis to steatohepatitis, ultimately culminating in irreversible liver damage [9].

With an estimated 38% of the world’s population affected, NAFLD has quickly risen to the top of the liver disease spectrum worldwide. Although only a small percentage of NAFLD patients progress to cirrhosis or hepatocellular cancer, the increasing number of individuals at risk of these serious outcomes is concerning. Particularly alarming is the trend of NAFLD manifesting at younger ages, extending the window for potential serious consequences [10]. Despite its widespread prevalence, NAFLD lacks specific pharmacological treatments. This deficiency in treatment options is attributed to NAFLD’s multifactorial nature, which is compounded by an incomplete understanding of its underlying mechanisms, a lack of precise and cost-effective imaging techniques, and an inadequacy of reliable non-invasive biomarkers [4]. Lately, the MedDiet has emerged as a recommended therapeutic approach for NAFLD patients as a suitable strategy to deter its onset and mitigate the progression to severe forms [11]. High adherence to the MedDiet has been correlated with a reduction in intrahepatic fat levels, together with improved MetS indicators. Consequently, heightened adherence to a Mediterranean dietary regimen may confer benefits beyond minimizing MetS and diabetes and potentially reversing fatty liver disease [12].

Erythrocytes are essential for oxygen transportation in the body. They lack a nucleus, possess remarkable flexibility, and have a lifespan of approximately 120 days. Physiological stress can increase the production of ROS, leading to oxidative stress that damages erythrocyte membranes. Due to their constant exposure to oxygen, erythrocytes have antioxidant defence mechanisms, including enzymes like catalase (CAT), superoxide dismutase (SOD), and glutathione peroxidase (GPx), to mitigate oxidative damage and maintain cellular integrity [13]. Novel biomarkers associated with oxidative stress are currently under research for their potential diagnostic and monitoring utility. There is a primary focus on analysing these biomarkers in blood samples due to their accessibility and ability to reflect tissue-level events. Erythrocytes, as fundamental constituents of the blood, are affected by circulating inflammatory mediators and oxidative stress, resulting in significant disruptions to cellular membrane integrity and function in various pathological conditions [14].

This study aimed to understand how the adherence of NAFLD participants to the MedDiet influenced oxidative stress and inflammatory biomarkers in plasma, erythrocytes, and peripheral blood mononuclear cells (PBMCs) after a 2-year intervention.

## 2. Methods

### 2.1. Study Design

This is a two-year prospective randomized trial conducted in Mallorca, Spain, aimed at evaluating the efficacy of a personalized nutritional intervention using a customized MedDiet and the promotion of physical activity. The study protocols adhered to the Declaration of Helsinki ethical principles, and the Ethics Committee of the Balearic Islands (CEIC-IB2251/14PI) authorized each step of the process. All participants provided their informed permission after being made aware of the study’s goals and potential outcomes. NCT04442620 is the registration number for this study on ClinicalTrials.gov https://clinicaltrials.gov/ct2/show/NCT04442620 (accessed on 22 June 2020) [15].

### 2.2. Participants

Forty adults, with women comprising 50% of the cohort, aged 40–60 years with a body mass index (BMI) ranging from 27 to 40 kg/m^2^, diagnosed with NAFLD by MRI, and meeting the MetS criteria according to the International Diabetes Federation (IDF) [16] were involved in this study. Exclusion criteria encompassed a history of cardiovascular disease, liver conditions other than NAFLD, recent cancer or malignancy within the past 5 years, hemochromatosis, prior bariatric surgery, untreated depression, substance abuse, pregnancy, primary endocrine disorders excluding untreated hypothyroidism, severe psychiatric disorders (such as schizophrenia, bipolar disorder, eating disorders, or recent hospitalization for depression within the last 6 months), a Beck Depression Inventory score exceeding 30, ongoing steroid therapy, or an inability to provide informed consent.

The participants’ adherence to the MedDiet pattern was evaluated using a validated questionnaire containing 17 items related to the diet. A higher score on this questionnaire showed greater adherence to the MedDiet [17]. Subsequently, individuals were divided into two groups depending on the level of enhancement in adherence to the MedDiet observed from the study baseline to the 2-year post-intervention. As a result, the two groups comprised individuals who showed substantial improvement in adherence, labelled ‘High Adherence’ (20 participants), and those who demonstrated lesser improvement, labelled ‘Low Adherence’ (20 participants), after 12 months, based on the scoring system.

### 2.3. Anthropometrics, Clinical Assessment, and Physical Activity

Accurate and consistent anthropometric measurements were conducted by trained professional dieticians who underwent identical and rigorous training to minimize the impact of interobserver coefficients of variation. Body weight, determined without shoes, was assessed using a Segmental Body Composition Analyzer (Tanita BC-418, Tanita, Tokyo, Japan), with a deduction of 0.6 kg for light clothing. Height measurements were taken with a mobile anthropometer (Seca 214, SECA Deutschland, Hamburg, Germany), ensuring the patient’s head was in the Frankfort Horizontal Plane position. BMI (kg/m^2^) was then calculated based on these measurements. Blood pressure was measured in triplicate while the patient was in a sitting position using a validated semi-automatic oscillometer (Omron HEM, 750CP, Hoofdrop, The Netherlands), and then the median value was calculated. Waist circumference, measured twice with an anthropometric tape midway between the final rib and the iliac crest, provided essential data. Additionally, the waist-to-height ratio (WHtR), recognized as a cardiovascular risk biomarker, was computed by dividing waist (in cm) by height (in cm).

Accelerometry (ActiGraph wGT3X-B; ActiGraph LLC, Pensacola, FL, USA) was used to quantify physical activity. The energy expenditure reported was calculated as the metabolic equivalent of a task (MET) minutes per day.

### 2.4. NAFLD Diagnosis

Intrahepatic fat contents (IFC) were performed with a 1.5-T Magnetic Resonance Imaging (MRI) (Signa Explorer 1.5T, General Electric Healthcare, Chicago, IL, USA) by using a 12-channel phased-array coil [18]. Abdominal MRI allows quantification of the liver fat as a mean percentage, and a mean IFC ≥ 6.4% was established as clinically relevant [19].

### 2.5. Blood Collection and Analysis

Venous blood samples were taken from the antecubital vein after an overnight fasting period, and subsequent biochemical analyses were conducted on glucose, glycated haemoglobin A1c (HbA1c), triglycerides, high-density lipoprotein cholesterol (HDL-chol), low-density lipoprotein cholesterol (LDL-chol), total cholesterol, aspartate aminotransferase (AST), alanine aminotransferase (ALT), gamma-glutamyl transferase (GGT), and C-reactive protein (CRP) concentrations using established enzymatic methods in the clinical laboratory of the University Hospital de Son Espases (Palma de Mallorca, Spain). Haematological parameters and cell counts were evaluated using an automated flow cytometer analyser, specifically the Technion H2 VCS system (Bayer, Frankfurt, Germany).

Two vacutainers with ethylenediaminetetraacetic acid (EDTA) as an anticoagulant were collected and carried on to the university laboratory. One was used to obtain plasma and erythrocyte samples; fresh blood was centrifugated at 1700× *g* 15 min at 4 °C. The other vacutainer was utilized to isolate peripheral blood mononuclear cells (PBMCs) as previously described [20]. In summary, 6 mL of blood was layered onto 4 mL of Ficoll–Paque PLUS reagent (GE Healthcare Bio-Sciences AB, Uppsala, Sweden) and centrifuged at 900× *g* for 30 min at 4 °C. The PBMC layer was collected and washed twice with phosphate-buffered saline (PBS) at pH 7.4, followed by centrifugation at 900× *g* for 10 min at 4 °C.

### 2.6. Antioxidant Determinations and Antioxidant Score

Antioxidant determinations in erythrocytes were assessed using a Shimadzu UV-2100 spectrophotometer (Shimadzu Corporation, Kyoto, Japan) at 37 °C. Catalase (CAT), superoxide dismutase (SOD), glutathione peroxidase (GPx), and glutathione reductase (GRd) were enzymatic antioxidants, and total glutathione (GSH) was a non-enzymatic antioxidant. CAT activity was analysed at 240 nm by Aebi’s method based on the decomposition of H_2_O_2_ [21]. SOD activity was determined by an adaptation of McCord and Fridovich’s method at 550 nm [22]. GPx and GRd activities were assessed at 339 nm using an adaptation of the Flohé and Günzler [23] and Goldberg and Spooner [24] methods, respectively. GSH was quantified at 412 nm employing Tietze’s method [25], which involves deproteinization of samples, neutralization prior to DTNB reduction, and reference to a calibration curve constructed with standard glutathione.

An antioxidant score was calculated for both time points, which were then used to determine the change in antioxidant score after 2 years of intervention. This calculation was performed as follows: First, quintiles were established for each biomarker both at baseline and after the 2-year intervention, using identical cut-offs (Table 1). Second, the sum of the quintiles of the five biomarkers was calculated at baseline and after the 2-year intervention, resulting in baseline and follow-up antioxidant scores. Finally, the difference between the baseline and post-intervention antioxidant scores was calculated. This score ranged from 5 (indicating placement in the first quintile for all biomarkers) to 25 (reflecting placement in the fifth quintile for all biomarkers). A normal distribution was observed for baseline values at 2 years, as well as for changes in antioxidant score.

### 2.7. Malondialdehyde Assay

Malonaldehyde as a marker of lipid peroxidation was analysed in plasma and in erythrocytes of all participants by a specific colorimetric assay kit (Sigma-Aldrich Merck^®^, St. Louis, MO, USA), where the absorbance was measured at 586 nm following the manufacturer’s instructions.

### 2.8. Immunoassay Kits

Cytokeratin 18 (CK-18) levels were estimated in plasma using a one-step in vitro immunoassay, M30 Apoptoense^®^ ELISA (PEVIVA^®^, in the USA, Canada, and Japan), and oxidized low-density lipoprotein (oxLDL) levels were determined in plasma by an ELISA kit (FineTest^®^, Wuhan, China). Immunoassay kits were utilized in accordance with the manufacturer’s instructions for comprehensive and accurate results and read at 450 mn.

### 2.9. ROS Production in PBMCs

Radical oxygen species (ROS) production in PBMCs was determined after activation with Zymosan A (Zym) (1 mg/mL PBS) from *Saccharomyces cerevisiae* (Sigma-Aldrich) and lipopolysaccharide (LPS) (100 μg/mL phosphate-buffered saline—PBS) from Escherichia coli (Sigma-Aldrich, St. Louis, MO, USA). A total of 50 μL of cell suspension (containing about 6 × 10^5^ cells) was added to a 96-well microplate, and 50 μL of LPS or Zym prepared in PBS was added to the wells. Finally, 2,7-dichlorofluorescein-diacetate (DCFH-DA, 61.6 μM in Hanks’ Balanced Salts Medium) as an indicator was added to all wells. Fluorescence (Ex, 480 nm; Em, 530 nm) was measured at 37 °C for 1 h with a FLx800 Microplate Fluorescence Reader (Biotek Instruments, Inc., Winuschi, VT, USA) by punctual ultraviolet light exposures, and emission readings were recorded every minute. ROS concentration was calculated by measuring the fluorescence of a standard curve of known ROS concentration after its reaction with DCFH-DA in the same conditions as the samples.

### 2.10. RNA Extraction and Real-Time PCR

The RNA extraction from PBMCs was carried out using Tripure^®^ (Tripure Isolation Reagent, Roche Diagnostics, Mannheim, Germany), following which 1 μg of RNA per sample underwent reverse transcription using TaqMan Reverse Transcription Reagents (Life Technologies^®^, Vall Allen Way Carlsbad, CA, USA) at 42 °C for 60 min, followed by 5 min at 99 °C, in a final volume of 10 μL, according to the manufacturer’s instructions. A total of 3 μL of the resultant cDNA was amplified using Light-Cycler^®^ 480 SYBR^®^ Green I Master (Roche Diagnostics, Mannheim, Germany). Target cDNAs were amplified using LightCycler^®^ 96 for 45 cycles following an initial denaturation step of 10 min at 95 °C. Real-time PCR was employed to measure the mRNA expression of CAT, manganese superoxide dismutase (MnSOD), and Toll-Like Receptor 4 (TLR4), utilizing human 18S ribosomal RNA as the reference gene and a fluorescent reporter dye. Table 2 lists the primer sequence and the conditions for amplification. The baseline values of patients achieving low adherence to the Mediterranean diet were designated as the reference group and assigned a designation of 1.

### 2.11. Statistical Analysis

Statistical analysis was carried out with the Statistical Package for Social Sciences (SPSS v.29, IBM Software Group, Chicago, IL, USA). Variables were presented as means ± standard deviation (SD), considering *p* < 0.05 as statistically significant. The Kolmogorov–Smirnov test was used to assess the normality of the data. A two-way analysis of covariance (ANCOVA) after adjustment for age, gender, and intervention group was used to check the significance of the data. A Bonferroni post hoc test was carried out when significant differences were found between groups.

## 3. Results

**Figure 1:**
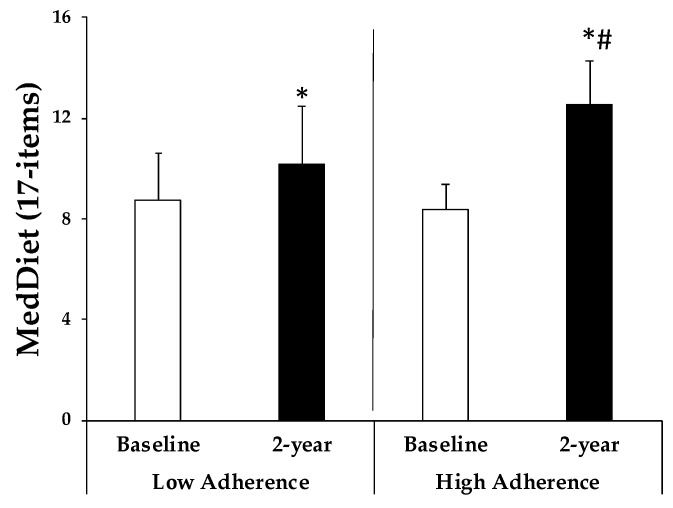
Adherence to Mediterranean Dietary (MedDiet) is expressed as points obtained in the questionnaire at baseline and 2-years stratified by adherence to MedDiet groups. Results are expressed as means ± SD. Two-way analysis of co-variance (ANCOVA) after adjustments by age, sex, and intervention (diet and physical activity). * Differences in means between participants in time (baseline and 2-year). # Differences in means between groups (low and high adherence). Data points are significant when *p* < 0.05.

The adherence to the MedDiet at both the study baseline and after 2 years of intervention by the participants is illustrated in Figure 1. It can be observed that in both groups, there was a significant increase in adherence to this MedDiet. However, the participants with higher adherence after the 2-year intervention demonstrated a significantly greater increase compared to those with lower adherence. At baseline, there were no differences between both groups, with adherence scores of 8.7 ± 1.9 and 8.3 ± 1.0 for low and high adherence, respectively. After the 2-year intervention, MedDiet adherence increased to 10.2 ± 2.3 and 12.5 ± 1.8, respectively.

The anthropometric, clinical, and haematological parameters of participants with NAFLD, categorized by their adherence to MedDiet after a 2-year intervention, are summarized in Table 3. Significant differences were evidenced in weight, BMI, cholesterol total, HDL-chol, LDL-chol, and triglycerides when comparing the evolution after a 2-year intervention in both groups. However, only significant differences were observed after 2-years of intervention in the high adherence group in glycemia, AST, ALT, GGT levels, and IFC. After a 2-year intervention, significant differences were found between groups in weight and BMI. Despite no significant changes in systolic blood pressure, participants who only slightly improved their adherence to the MedDiet experienced an increase in diastolic blood pressure, while those with higher adherence appeared to decrease it, albeit not significantly. Regarding haematological parameters, at baseline, participants stratified as having low adherence exhibited a low haematocrit and higher neutrophil levels compared to participants with high adherence. After a 2-year intervention, participants with high adherence experienced a decrease in their erythrocyte levels. No differences were observed in the remaining clinical, haematological, and physical activity paramaters. The number of individuals using antihypertensives (37% of participants in the Low Adherence to MedDiet group and 37% of participants in the High Adherence to MedDiet group), hypoglycaemic medicines (5% of participants in the Low Adherence to MedDiet group and 0% of participants in the High Adherence to MedDiet group), and hypolipidemic medications (32% of participants in the Low Adherence to MedDiet group and 16% of participants in the High Adherence to MedDiet group) was similar in both groups, according to the statistical analysis of the data. This suggests that medication does not appear to significantly influence the improvement of IFC with lifestyle intervention.

**Figure 2:**
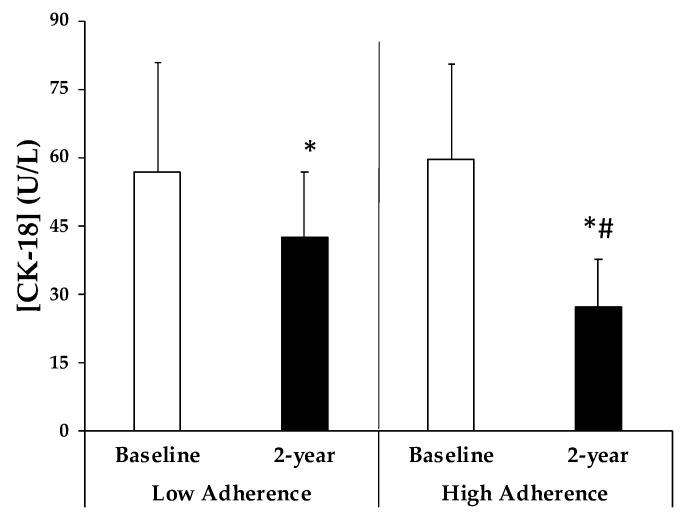
Cytokeratin 18 (CK-18) plasma levels at baseline and 2-years, stratified by adherence to MedDiet. Results are expressed as means ± SD. Two-way analysis of co-variance (ANCOVA) after adjustments by age, sex, and intervention (diet and physical activity). * Differences in means between participants in time (baseline and 2-year). # Differences in means between groups (low and high adherence). Data points are significant when *p* < 0.05.

The plasma levels of CK-18, a biomarker of liver health, are illustrated at the study baseline and after a 2-year intervention (Figure 2). It can be observed that in both groups, there was a significant decrease in their levels after a 2-year intervention. However, the group that achieved higher adherence to the MedDiet showed significantly lower CK-18 plasma levels after a 2-year intervention compared to the group with lower adherence.

Table 4 displays the enzymatic antioxidant activities of CAT, SOD, GPx, and GRd, as well as GSH levels in erythrocytes. All these antioxidants showed significantly higher levels after a 2-year intervention in the group with higher adherence to the MedDiet compared to baseline, as well as compared to the lower adherence group. GPx activity also increased in participants with low adherence to the MedDiet. OxLDL plasma levels and MDA plasma and erythrocyte levels were reduced in participants with high adherence to the Mediterranean Diet after a 2-year intervention. Erythrocyte MDA levels were lower after 2 years in the group with high adherence compared to the group with low adherence.

CAT, MnSOD, TLR4, GPx, and GRd relative mRNA expressions were assessed in PBMCs (Table 4). The mRNA levels of CAT and TLR4 showed a significant increase after the 2-year intervention in the group with high adherence compared to baseline. No differences were found in MnSOD, GPx, or GRd gene expression.

Significant negative correlations were observed between adherence to the MedDiet modification and changes in weight, BMI, IFC, glycemia, AST, and erythrocyte levels (Table 5). As adherence to the Mediterranean diet increased, a decrease in the parameters was registered.

**Figure 3:**
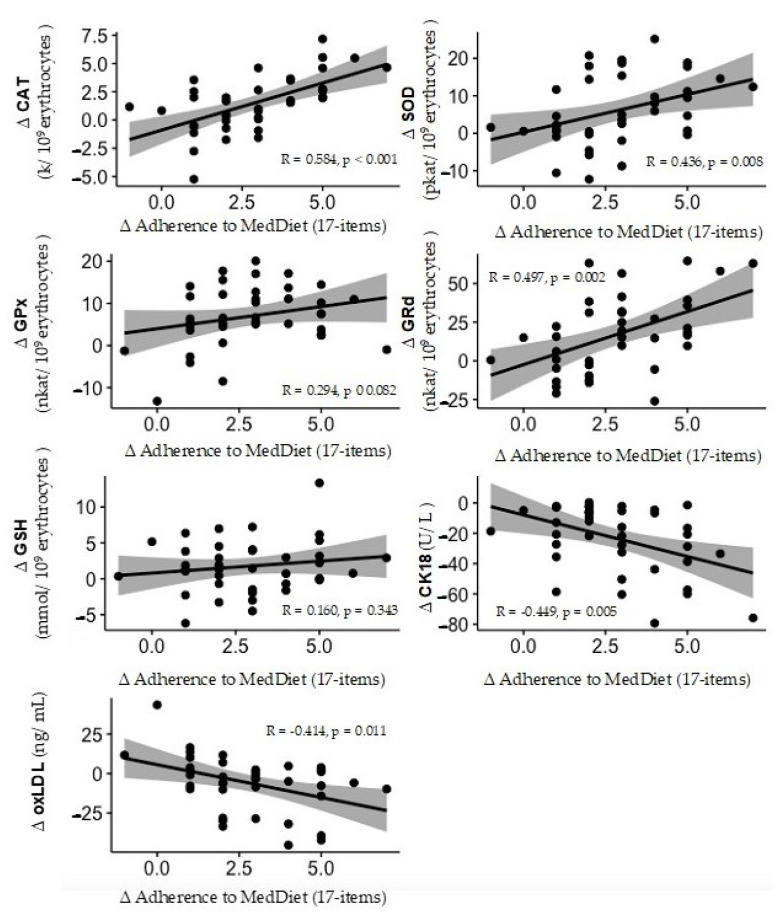
Correlation between adherence to the Mediterranean Diet (MedDiet) and antioxidants, CK-18, and oxLDL change levels after 2 years of lifestyle intervention. Statistical analysis: bivariate correlation test adjusted by age, sex, and the intervention (diet and physical activity).

In Figure 3, correlations between adherence to the MedDiet and antioxidants, CK-18, and oxLDL change levels after a 2-year lifestyle intervention are depicted. Negative correlations were found between changes in oxLDL and CK-18 levels and adherence to the MedDiet, while positive correlations were found between changes in CAT, SOD, and GRd activities.

The linear regression analysis of the relationship between changes in adherence to the Mediterranean Diet and changes in antioxidant score has a b-standardized coefficient of 0.659 and *p* < 0.001 (Table 6).

## 4. Discussion

The primary outcomes of this study reveal a strong correlation between improvements in adherence to the MedDiet after the 2-year intervention and improved oxidative stress biomarkers in erythrocytes in NAFLD patients, suggesting potential therapeutic implications. Participants with higher adherence to the MedDiet demonstrated a more favourable disease trajectory, marked by reductions in IFC, and plasma levels of CK-18 and oxLDL. These improvements in oxidative stress biomarkers underscore the potential therapeutic benefits of adhering closely to the MedDiet in managing NAFLD and mitigating its progression. The beneficial health effects of the MedDiet can be attributed to a combination of factors such as low energy density, a balanced proportion of essential and heart-healthy fatty acids, high amounts of dietary fibre, and an array of bioactive compounds with antioxidant and anti-inflammatory properties [26]. Indeed, many studies have evidenced that dietary bioactive compounds, such as polysaccharides, polyphenols, isoflavones, and alkaloids, hold promise in ameliorating NAFLD and other metabolic disorders, including diabetes, MetS, and menopause [27,28]. Since the present intervention is focused on promoting adherence to the MedDiet, it is plausible that the bioactive compounds present in certain foods consumed by participants may have contributed to the beneficial effects observed in reducing oxidative stress and other biomarkers related to NAFLD.

All participants exhibited weight reduction regardless of their adherence to the MedDiet at the 2-year intervention. However, it is noteworthy that participants who successfully enhanced their adherence to the MedDiet achieved higher weight loss and BMI reduction than those who did not. These findings are consistent with previous studies demonstrating the favourable effects of MedDiet adherence on NAFLD, even in the absence of significant caloric restriction. Specifically, improvements were observed with just a minimal weight loss of 2%. However, when MedDiet interventions were combined with caloric restriction, more substantial weight loss occurred. Thus, combining caloric restriction with adherence to the MedDiet may represent an effective strategy for addressing NAFLD [11].

The current results demonstrate that both groups achieved an improved lipid profile characterized by decreases in total cholesterol, LDL-chol, and triglyceride levels, along with an increase in HDL-chol levels. These outcomes are in accordance with a previous meta-analysis that suggests the beneficial effects of the MedDiet on lipid profiles (total cholesterol, LDL-chol, and triglycerides), blood pressure, insulin resistance, levels of inflammation biomarkers, and reduced blood glycemia levels as compared to the control diet in NAFLD patients [29]. However, it is noteworthy that only participants who achieved higher adherence to the MedDiet also experienced reductions in glycemia levels and liver enzymes such as AST, ALT, and GGT. Given that these enzymes serve as primary markers of liver dysfunction, these results demonstrate how higher adherence to the MedDiet can significantly reduce hepatic damage. Despite the existence of unconclusive results in the current literature, the latest Systematic Review and Meta-Analysis of Randomized Control Trials published concludes that the MedDiet’s impact on NAFLD highlights the considerable potential of this dietary pattern in ameliorating parameters associated with NAFLD severity, including improvement in liver function enzymes and NAFLD scores [30]. In agreement with previous studies, IFC was more reduced in participants with high adherence to MedDiet than in those with low adherence [12,31].

In the context of haematological parameters, discernible discrepancies were noted solely within erythrocyte cells following a two-year intervention period among patients exhibiting high adherence to the MedDiet. While there is a lack of literature directly correlating MedDiet with erythrocyte counts, heightened adherence to the MedDiet was associated with a decrease in erythrocyte levels. Elevated erythrocyte counts have been previously linked to an increased risk of both the incidence and progression of NAFLD [32]. Although the exact mechanism underlying the relationship between erythrocyte levels and NAFLD remains unclear, our findings suggest that adherence to the MedDiet may influence erythrocyte counts, potentially impacting the pathogenesis and progression of NAFLD. Further research is warranted to elucidate the mechanistic link between the MedDiet, erythrocyte levels, and NAFLD outcomes.

CK-18 is released into the circulation upon hepatocyte damage, rendering it a valuable a biomarker of disease progression in NAFLD and liver injury. Significantly high levels of CK-18 were correlated with a high percentage of IFC in patients diagnosed with NAFLD via MRI [33]. The reduction in CK18 levels was observed in both groups, albeit more pronounced in the group with higher adherence to the Mediterranean diet. Thus, it can be supposed that higher adherence to the Mediterranean diet is associated with lower plasma CK-18 levels and ameliorated hepatocyte damage.

As the primary defence against ROS in biological systems, CAT, SOD, and GPx are recognized as the most potent antioxidant enzymes within cells [34]. Following a 2-year intervention, participants who demonstrated higher adherence to the MedDiet exhibited enhanced antioxidant activity levels compared to those with lower adherence. Additionally, there was an increase in the relative expression of CAT in PBMCs from the group’s higher adherence to the MedDiet. Oxidative stress may also have an adverse effect on antioxidant defence systems. Overproduction of ROS can directly inhibit the activities of antioxidant enzymes, such as SOD and CAT, or deplete antioxidant molecules, such as GSH [35]. These findings suggest that increased adherence to the MedDiet, rich in antioxidants such as polyphenols, polyunsaturated fatty acids, flavonoids, and terpenoids, promotes higher activity of endogenous antioxidants in erythrocytes among NAFLD patients. Moreover, significant correlations were observed between higher adherence to MedDiet and increased activities of CAT, SOD, and GRd in erythrocytes. This further underscores the potential of the MedDiet to enhance antioxidant defence mechanisms and mitigate oxidative stress in individuals with NAFLD.

In NAFLD patients, heightened ROS production from pre-activated circulating immune cells and disrupted lipid metabolism contribute to oxidative damage, thereby exacerbating liver injury [36]. The current study demonstrates a reduction in ROS production by PBMCs activated with zymosan and LPS only in participants with a high adherence to MedDiet after a 2-year-follow-up, possibly reflecting a decrease in pro-oxidative status. Both zymosan and LPS act as microbial activators that engage TLRs, crucial components of the innate immune system. PBMC activation by LPS involves interaction with TLR4, while zymosan interacts with TLR2/6, ultimately leading to NADPH oxidase activation [37]. Moreover, bacterial endotoxins have been reported to activate TLRs on liver cells, resulting in NF-κb and inflammasome activation. Furthermore, endotoxins directly damage hepatocytes and stimulate Kupffer cells, triggering the release of inflammatory cytokines and oxygen radicals, exacerbating inflammation [38]. These findings are consistent with a reduction in TLR4 expression observed mainly in the high-adhered group, suggesting that MedDiet compounds can contribute to reducing the prooxidative and proinflammatory states present in NAFLD patients.

MDA is a well-established indicator of oxidative stress, reflecting the peroxidation of polyunsaturated fatty acids by ROS [39]. In the current study, plasma MDA levels were reduced in both groups after increasing their adherence to MedDiet, while erythrocyte MDA levels were only reduced in participants with higher adherence. These outcomes highlight how NAFLD patients, following an intervention based on the MedDiet, effectively reduced their oxidative stress markers in both blood samples. In this sense, the current results are in line with previous studies reporting that normal-weight participants consuming a high-quality diet experienced an increase in total antioxidant capacity and a decrease in erythrocyte MDA levels [40]. Furthermore, a meta-analysis suggested that high polyphenol olive oil, a component of the MedDiet, may contribute to the reduction of MDA and oxLDL levels, adding further support to the cardioprotective effects of this dietary pattern [41]. OxLDL molecules are recognized as crucial pathogenic determinants in vascular atherosclerosis. The development of portal venous atherosclerosis, triggered by the accumulation of free cholesterol and oxLDL, represents a distinctive characteristic of NAFLD [42]. In our study, oxLDL levels were significantly reduced in the group of participants with higher adherence to the MedDiet. Similar results were previously observed in a cohort of subjects with MetS following a traditional MedDiet for 1 year [43]. Moreover, MedDiet is associated with high improvements in cardiovascular disease risk factors in women with MetS and high LDL-chol, as evidenced by notable decreases in oxLDL, atherogenic lipoprotein subfractions, and LDL-chol [44].

Finally, the current findings from a linear regression analysis demonstrate that as adherence to the Mediterranean diet increases, there is a corresponding rise in the antioxidant score.

Recent research underscores the close relationship between the composition of the intestinal microbiome and the development of NAFLD. This anatomical and physiological connection between the liver and the intestine facilitates bidirectional communication between the intestinal microbiome and its metabolites, which may significantly influence the pathogenesis of NAFLD [45]. Therefore, considering the assessment of microbiome composition in future research could provide a more comprehensive understanding of the underlying mechanisms of the disease and potential therapeutic targets. It is well known that the MedDiet pattern is rich in anti-inflammatory and antioxidant compounds. In fact, several studies showed that, in addition to following this type of diet and adopting healthy and active lifestyles, natural active compounds are beneficial for NAFLD in the constellation of risk factors that characterize MetS [27,46].

## 5. Strengths and Limitations

Although there are many studies that relate the impact of the MedDiet on NAFLD, the current study significantly contributes to advancing understanding in the field by examining the impact of the MedDiet on IFC and levels of oxidative stress biomarkers in erythrocytes over a 2-year intervention period. The longitudinal approach employed in this study offers more robust evidence compared to cross-sectional methodologies, while adherence to a standardized protocol helps mitigate the risk of bias. Moreover, current findings hold practical significance for clinical application, particularly given the absence of alternative treatments for NAFLD at present. However, it is important to acknowledge several limitations. Firstly, the relatively small sample size undermines the confidence of current conclusions, highlighting the need for larger cohorts. Secondly, the age range (40–60 years) of participants restricts the generalizability of current findings to broader populations. Thirdly, the initial classification of groups based on diet adherence introduces a selection bias, leading to some loss of homogeneity. Nevertheless, these constraints can be mitigated through the application of statistical analyses that consider variables such as age, sex, and the specific intervention group to which each participant was randomly assigned.

## 6. Conclusions

The current findings underscore a significant association between increased adherence to the MedDiet over a 2-year period and improved oxidative stress biomarkers among NAFLD patients, suggesting potential therapeutic benefits. Participants with higher adherence to the MedDiet exhibited more favourable disease progression, including decreases in insulin resistance, plasma circulating levels of CK-18, and oxLDL, along with decreased levels of prooxidant biomarkers and increased antioxidant activity in erythrocytes. These findings highlight the potential therapeutic impact of dietary interventions on NAFLD management. By targeting oxidative stress and associated pathways, dietary modifications may offer promising avenues for improving liver health and mitigating the progression of NAFLD.

## Figures and Tables

**Table 1:** Cut-off points used to create the antioxidant score at baseline and at follow-up.

Antioxidants	Quintiles	1	2	3	4	5
CAT (k/10^9^ erythrocytes)	Cut-off points	<8.78	8.78–10.25	10.25–10.96	10.97–12.19	>12.19
SOD (pkat/10^9^ erythrocytes)		<24.96	24.96–28.26	28.26–32.65	32.65–37.32	>37.32
GPx (nkat/10^9^ erythrocytes)		<43.87	43.86–49.40	49.40–52.66	52.66–57.08	>57.08
GRd (nkat/10^9^ erythrocytes)		<96.73	96.73–106.52	106.52–119.29	119.29–130.09	>130.09
GSH (mmol/10^9^ erythrocytes)		<4.69	4.69–6.12	6.12–7.07	7.07–9.13	>9.13

Abbreviations: CAT: catalase, SOD: superoxide dismutase, GPx: glutathione peroxidase, GRd: glutathione reductase, and GSH: total glutathione.

**Table 2:** Primers and conditions used in real-time PCRs.

Gene	Primer	Conditions	
18S	Fw: 5′-ATgTgAAgTCACTgTgCCAg Rv: 5′-gTgTAATCCgTCTCCACAgA	95 °C 60 °C 72 °C	10 s 10 s 15 s
CAT	Fw: 5′-TTTggCTACTTTgAggTCAC Rv: 5′-TCCCCATTTgCATTAACCAg	95 °C 60 °C 72 °C	10 s 10 s 15 s
MnSOD	Fw: 5′-CgTgCTCCCACACATCAATC Rv: 5′-TgAACgTCACCgAggAgAAg	95 °C 60 °C 72 °C	10 s 10 s 12 s
TLR4	Fw: 5′-ggTCACCTTTTCTTgATTCCA Rv: 5′-TCAgAggTCCATCAAACATCAC	95 °C 60 °C 72 °C	10 s 10 s 15 s
GPx	Fw: 5′-TTCCCgggCAACCAgTTTg Rv: 5’-TTCACCTCTCACTTCTCgAA	95 °C 63 °C 72 °C	10 s 10 s 15 s
GRd	Fw: 5′-TCACgCAgTTACCAAAAggAAA Rv: 5′-CACACCCAAgTCCCCTgCATAT	95 °C 63 °C 72 °C	10 s 10 s 15 s

Abbreviations: 18S: human 18S ribosomal, CAT: catalase, MnSOD: manganese superoxide dismutase, TLR4: Toll-Like Receptor 4, GPx: glutathione peroxidase, and GRd: glutathione reductase.

**Table 3:** Characteristics of participants according to their adherence to the Mediterranean Diet (MedDiet) at baseline and after 2-year intervention.

	Low Adherence to MedDiet		High Adherence to MedDiet		*p*-Value
	Baseline (*n* = 20)	2-Year Change (*n* = 20)	Baseline (*n* = 20)	2-Year Change (*n* = 20)	
Anthropometry					
Weight (kg)	88.8 ± 9.94	87.6 ± 10.2 *	87.2 ± 9.1	81.7 ± 8.9 *#	<0.001
BMI (kg/m^2^)	32.6 ± 2.1	32.1 ± 2.2 *	32.2 ± 2.3	30.2 ± 1.9 *#	<0.001
Systolic BP (mmHg)	135.3 ± 17.3	136.1 ± 14.6	136.6 ± 17.7	134.8 ± 21.3	0.536
Diastolic BP (mmHg)	82.0 ± 8.0	85.6 ± 10.8 *	81.7 ± 9.5	83.8 ± 11.1	0.345
Clinical parameters					
Glucose (mg/dL)	109.8 ± 19.6	102.2 ± 14.7	109.4 ± 21.3	97.1 ± 15.1 *	0.275
HbA1c (%)	6.0 ± 0.8	5.9 ± 0.7	5.96 ± 1.0	5.8 ± 0.7	0.642
Cholesterol total (mg/dL)	198.3 ± 35.0	186.9 ± 30.6 *	208.4 ± 26.5	186.8 ± 22.0 *	0.094
HDL-c (mg/dL)	41.9 ± 8.6	45.7 ± 13.1 *	39.9 ± 7.4	44.4 ± 9.3 *	0.806
LDL-c (mg/dL)	129.7 ± 30.5	119.1 ± 28.0 *	134.1 ± 34.9	118.6 ± 29.1 *	0.433
Triglycerides (mg/dL)	172.8 ± 43.3	153.6 ± 40.6 *	179.8 ± 27.4	145.2 ± 32.5 *	0.197
CRP (mg/dL)	0.6 ± 0.5	0.4 ± 0.4	0.5 ± 0.7	0.4 ± 0.5	0.617
AST (U/L)	26.5 ± 6.9	24.6 ± 6.9	27.4 ± 7.3	21.1 ± 6.0 *	0.014
ALT (U/L)	36.5 ± 18.2	33.2 ± 18.1	37.0 ± 12.4	28.0 ± 7.7 *	0.094
GGT (U/L)	7.8 ± 7.8	32.6 ± 9.2	37.8 ± 11.6	30.7 ± 10.7 *	0.075
IFC (%)	16.1 ± 4.6	14.3 ± 4.5	16.6 ± 4.7	12.1 ± 5.3 *	0.012
Haematological parameters					
Haematocrit (%)	42.9 ± 4.4	42.7 ± 4.1	44.5 ± 4.2 #	44.2 ± 3.6	0.299
Erythrocytes (10^6^/μL)	4.9 ± 0.4	4.8 ± 0.4	4.9 ± 0.4	4.8 ± 0.36 *	0.332
Leukocytes (10^3^/μL)	7.6 ± 1.6	7.2 ± 1.1	7.4 ± 2.0	7.4 ± 2.3	0.125
Platelets (10^3^/μL)	245.1 ± 40.5	237.8 ± 49.4	236.7 ± 38.4	228.3 ± 37.7	0.705
Neutrophils (10^3^/μL)	4.4 ± 1.0	4.02 ± 0.9	3.9 ± 1.3	3.9 ± 1.6	0.077
Lymphocytes (10^3^/μL)	2.3 ± 0.6	2.3 ± 0.5	2.7 ± 0.7	2.7 ± 0.6	0.452
Monocytes (10^3^/μL)	0.6 ± 0.1	0.5 ± 0.1	0.6 ± 0.1	0.6 ± 0.2	0.198
Eosinophils (10^3^/μL)	0.2 ± 0.1	0.2 ± 0.1	0.2 ± 0.1	0.2 ± 0.1	0.916
Basophils (10^3^/μL)	0.1 ± 0.0	0.1 ± 0.0	0.1 ± 0.0	0.1 ± 0.0	0.334
Energy Expendidure					
Measured accelerometer (MET/day)	1850 ± 243	1802 ± 237	1862 ± 342	1795 ± 230	0.915

Abbreviations: MedDiet: Mediterranean Diet; BMI: body mass index, systolic BP: systolic blood pressure; diastolic BP: diastolic blood pressure, HbA1c: glycated haemoglobin A1c, HDL-chol: high-density lipoprotein, LDL-chol: low-density lipoprotein, AST: aspartate aminotransferase, ALT: alanine aminotransferase, GGT: gamma glutamyl transferase, CRP: c-reactive protein, IFC: intrahepatic fat content, SD: standard deviation. Results are expressed as means ± SD. Two-way analysis of co-variance (ANCOVA) after adjustments by age, sex, and intervention (diet and physical activity). * Differences in means between participants in time (baseline and 2-year). # Differences in means between groups (low and high adherence). Data points are significant when *p* < 0.05.

**Table 4:** Oxidative stress biomarkers in erythrocytes and plasma according to their adherence to the Mediterranean Diet (MedDiet) at baseline and after 2-year intervention.

	Low Adherence to MedDiet		High Adherence to MedDiet		*p*-Value
	Baseline (*n* = 20)	2-Year Change (*n* = 20)	Baseline (*n* = 20)	2-Year Change (*n* = 20)	
Antioxidants					
CAT (k/10^9^ erythrocytes)	10.0 ± 1.9	10.0 ± 1.6	9.8 ± 1.9	12.6 ± 1.8 *#	<0.001
SOD (pkat/10^9^ erythrocytes)	26.8 ± 7.5	28.4 ± 3.0	27.8 ± 8.4	38.0 ± 2.8 *#	0.002
GPx (nkat/10^9^ erythrocytes)	46.2 ± 6.0	50.4 ± 8.3 *	48.2 ± 7.6	57.8 ± 6.2 *#	0.018
GRd (nkat/10^9^ erythrocytes)	105.0 ± 15.3	109.4 ± 16.3	104.8 ± 21.2	134.0 ± 13.5 *#	0.001
GSH (mmol/10^9^ erythrocytes)	5.8 ± 2.7	6.8 ± 1.9	6.0 ± 2.3	8.5 ± 3.2 *#	0.240
PBMCs mRNA expression					
CAT (%)	100.0 ± 105.7	148.7 ± 35.0	112.4 ± 32.5	191.5 ± 113.8 *	0.005
MnSOD (%)	100.0 ± 123.8	150.6 ± 124.1	131.5 ± 72.7	181.3 ± 109.2	0.665
TLR4 (%)	100.0 ± 87.9	76.5 ± 103.9	116.5 ± 91.7	58.7 ± 35.1 *	0.024
GPx (%)	100.0 ± 176.6	120.7 ± 153.4	129.8 ± 162.5	156.1 ± 218.2	0.632
GRd (%)	100.0 ± 70.3	58.3 ± 51.3	86.9 ± 65.9	107.7 ± 90.8	0.132
Oxidative damage					
MDA (ng/10^9^ erythrocytes)	1.6 ± 0.4	1.5 ± 0.4	1.6 ± 0.4	1.1 ± 0.2 *#	0.003
MDA (ng/L plasma)	1.8 ± 0.6	0.1 ± 0.1 *	1.9 ± 0.7	0.2 ± 0.1 *	0.843
oxLDL (ng/mL)	34.0 ± 17.4	31.8 ± 8.7	35.4 ± 24.1	25.3 ± 13.5 *	0.097
ROS production in PBMCs stimulated with zymosan (RLU/min·10^3^ cells)	2199 ± 1757	1054 ± 476	2887 ± 1402	1022 ± 514 *	0.481
ROS production in PBMCs stimulated with LPS (RLU/min·10^3^ cells)	802.8 ± 1017	1702 ± 1960	451.4 ± 345.8	385.4 ± 329.8 *	0.213

Abbreviations: MedDiet: Mediterranean Diet; CAT: catalase, SOD: superoxide dismutase, GPx: glutathione peroxidase, GRd: glutathione reductase, GSH: total glutathione, MDA: malondialdehyde, oxLDL: oxidized low-density lipoprotein, MnSOD: manganese superoxide dismutase, TLR4: Toll-like receptor 4. Results are presented as means ± SD. Two-way analysis of co-variance (ANCOVA) after adjustments by age, sex, and intervention (diet and physical activity). * Difference in means between participants in time (baseline and 2-year). # Difference in means between groups (low and high adherence). Data points are significant when *p* < 0.05.

**Table 5:** Correlation between adherence to MedDiet and other biomarkers change after 2 years of lifestyle intervention.

		*p*-Value
Anthropometry		
Δ Weight (kg)	−0.638	<0.001
Δ BMI (kg/m^2^)	−0.629	<0.001
Δ Systolic BP (mmHg)	−0.163	0.336
Δ Diastolic BP (mmHg)	−0.194	0.250
Clinical parameters		
Δ Glycemia (mg/dL)	−0.399	0.014
Δ HbA1c (%)	−0.255	0.128
Δ Cholesterol total (mg/dL)	−0.220	0.191
Δ HDL-chol (mg/dL)	−0.053	0.756
Δ LDL-chol (mg/dL)	−0.118	0.488
Δ Triglycerides (mg/dL)	−0.146	0.389
Δ CRP (mg/dL)	−0.032	0.852
Δ AST (U/L)	−0.329	0.046
Δ ALT (U/L)	−0.234	0.163
Δ GGT (U/L)	−0.251	0.135
Δ IFC (%)	−0.364	0.027
Haematological parameters		
Δ Haematocrit (%)	−0.019	0.242
Δ Erythrocytes (10^6^/μL)	−0.326	0.049
Δ Leukocytes (10^3^/μL)	0.283	0.090
Δ Platelets (10^3^/μL)	0.000	0.999
Δ Neutrophils (10^3^/μL)	0.274	0.100
Δ Lymphocytes (10^3^/μL)	0.010	0.952
Δ Monocytes (10^3^/μL)	0.273	0.102
Δ Eosinophils (10^3^/μL)	0.150	0.375
Δ Basophils (10^3^/μL)	0.186	0.271
Oxidative damage		
Δ MDA (ng/10^9^ erythrocytes)	−0.244	0.145
Δ MDA (ng/L plasma)	−0.042	0.877

Abbreviations: BMI: body mass index, systolic BP: systolic blood pressure; diastolic BP: diastolic blood pressure, HbA1c: glycated haemoglobin A1c, HDL-chol: high-density lipoprotein, LDL-chol: low-density lipoprotein, AST: aspartate aminotransferase, ALT: alanine aminotransferase, GGT: gamma glutamyl transferase, CRP: c-reactive protein, IFC: intrahepatic fat content, MDA: malondyaldheide, CAT: catalase, MnSOD: manganese superoxide dismutase, TLR4: Toll-like eceptor 4, GPx: glutathione peroxidase, and GRd: glutathione reductase. Statistical analysis: bivariate correlation test adjusted by age, sex, and the intervention (diet and physical activity).

**Table 6:** Linear regression analysis of the relationship between changes in adherence to the MedDiet and changes in antioxidant score.

	b	SE b	b Standardized
Constant	−0.504	1.924	
Age	0.045	0.037	0.165
Sex	0.033	0.444	0.009
Intervention group	−0.342	0.311	−0.148
Δ Antioxidant Score	0.238	0.046	0.659 *

Statistical analysis: linear regression model adjusted by age, sex, and intervention (diet and physical activity). * *p* < 0.001.

## Data Availability

There are restrictions on the availability of data for this trial due to the signed consent agreements around data sharing, which only allow access to external researchers for studies following the project purposes. Requestors wishing to access the trial data used in this study can make a request to pep.tur@uib.es.
